# Managing household income and antiretroviral therapy adherence among people living with HIV in a low-income setting: a qualitative data from the HPTN 071 (PopART) trial in South Africa

**DOI:** 10.1186/s12981-023-00549-5

**Published:** 2023-08-04

**Authors:** Abenathi Mcinziba, Peter Bock, Graeme Hoddinott, Janet Seeley, Virginia Bond, Sarah Fidler, Lario Viljoen

**Affiliations:** 1 Desmond Tutu TB Centre, Department of Paediatrics and Child Health, Faculty of Medicine and Health Sciences, Stellenbosch University, Cape Town, South Africa; 2 School of Medicine, University of Zambia, Ridgeway Campus, Zambart, Lusaka, Zambia; 3 Department of Global Health and Development, London School of Hygiene and Tropical Medicine, London, UK; 4 Department of Medicine, Imperial College, London, UK

**Keywords:** ART adherence, HIV care, HPTN 071 (PopART), Household income, PLHIV

## Abstract

**Background:**

South Africa is reported to have the highest burden of HIV with an estimated 8.2 million people living with HIV (PLHIV) in 2021- despite adopting the World Health Organisation (WHO) universal HIV test and treat (UTT) recommendations in 2016. As of 2021, only an estimated 67% (5.5 million) of all PLHIV were accessing antiretroviral therapy (ART), as per recorded clinic appointments attendance. Studies in sub-Saharan Africa show that people living in low-income households experience multiple livelihood-related barriers to either accessing or adhering to HIV treatment including lack of resources to attend to facilities and food insecurity. We describe the interactions between managing household income and ART adherence for PLHIV in low-income urban and semi-urban settings in the Western Cape, South Africa.

**Methods:**

We draw on qualitative data collected as part of the HPTN 071 (PopART) HIV prevention trial (2016 – 2018) to provide a detailed description of the interactions between household income and self-reported ART adherence (including accessing ART and the ability to consistently take ART as prescribed) for PLHIV in the Western Cape, South Africa. We included data from 21 PLHIV (10 men and 11 women aged between 18 and 70 years old) from 13 households. As part of the qualitative component, we submitted an amendment to the ethics to recruit and interview community members across age ranges. We purposefully sampled for diversity in terms of age, gender, and household composition.

**Results:**

We found that the management of household income interacted with people’s experiences of accessing and adhering to ART in diverse ways. Participants reported that ART adherence was not a linear process as it was influenced by income stability, changing household composition, and other financial considerations. Participants reported that they did not have a fixed way of managing income and that subsequently caused inconsistency in their ART adherence. Participants reported that they experienced disruptions in ART access and adherence due to competing household priorities. These included difficulties balancing between accessing care and/or going to work, as well as struggling to cover HIV care-related costs above other basic needs.

**Conclusion:**

Our analysis explored links between managing household income and ART adherence practices. We showed that these are complex and change over the course of treatment duration. We argued that mitigating negative impacts of income fluctuation and managing complex trade-offs in households be included in ART adherence support programmes.

## Introduction

South Africa is reported to have the highest burden of HIV with an estimated 8.2 million people living with HIV (PLHIV) in 2021 [1]. Based on evidence that antiretroviral therapy (ART) started at any stage of HIV disease was beneficial to the individual person, the World Health Organisation (WHO) recommended that ART be made available to all PLHIV for life [2]. The South African Department of Health (NDoH) adopted these guidelines in 2016 [3, 4]. Since then, HIV/ART programmes in South Africa have continued to expand their services in order to facilitate the adherence of ART for PLHIV.

ART adherence involves the patient’s long-term and continued engagement with health care providers, pharmacy pill refills, taking medication daily at prescribed times, and following dietary instructions (meaning taking medication with/after/before meals as prescribed) [5, 6]. However, achieving optimal adherence to ART and long-term maintenance of treatment among PLHIV remains challenging. Based on facility records, it is estimated that in 2021, approximately 67% (5.5 million) of PLHIV were accessing ART in South Africa although adherence is difficult to track [1, 7]. There are noted challenges to conventional ART adherence monitoring and tracking in South Africa. Current ART adherence tracking is premised on patients’ self-reported adherence behaviour (which can be unreliable) and verified by blood viral load measurement (which is costly and not available in real-time) [4, 8, 9]. As a result, objective point-of-care devices to test and track ART adherence in real-time are being trialled in South Africa [10, 11].

Studies in sub-Saharan Africa found that PLHIV often experience financial barriers leading to disruption in adherence [9, 12]. A lack of transport fees to attend clinic visits, and financial and/or employment responsibilities which leads to missing clinic appointments have been found to negatively impact PLHIV’s ability to access ART [9, 13–15]. In addition, qualitative data from multiple studies across sub-Saharan Africa showed that PLHIV living in households with suboptimal income indicated that food insecurity contributes to inconsistent ART use, as ART side effects are aggravated when medication is taken without food [12, 16, 17].

In South Africa, all PLHIV have access to government provided ART services, free of direct costs [3]. However, inadequate household income still compromises the ART adherence of PLHIV. HIV care-related costs can still be incurred by PLHIV through indirect expenses such as buying healthy dietary foods, purchasing supplementary medication to ease the side effects from taking antiretroviral drugs (ARVs), and transport fees to preferred clinics for people who choose to access ART services outside of their community [9, 16, 18, 19].

We do not know how decisions around managing income interacts with ART access and the ability of PLHIV to adhere to ART. In this study, we draw on qualitative data collected as part of the HPTN 071 (PopART) study (a community randomised trial of a universal HIV test and treat strategy on HIV incidence) to understand how income and resources are managed in households affected by HIV [20]. We explore how decisions and management of income interacted with ART adherence of PLHIV in the Western Cape, South Africa.

## Methods

### Study design and setting

The HPTN 071 (PopART) trial was conducted in 21 communities, 12 in Zambia and 9 in South Africa, from 2013 to 2018. These study communities were randomly allocated to one of three study arms – Arms A, B, and C. Arms A and B received an HIV combination prevention package delivered at the household level with ART provided at government clinics [20, 21]. In Arm A and B communities, HIV education, testing, and referral to care were offered at the household level. Community members living with HIV in Arm A communities were eligible for ART regardless of their CD4 count, prior to national guideline changes, while people in Arm B communities received the household package but only started ART as per national guidelines by CD4 count threshold. People living in Arm C communities received HIV testing and treatment in accordance with the standard of care with no additional interventions [20]. Nested was a qualitative study that enrolled a separate cohort [21, 22]. Participants in the trial cohort who received regular visits from the study staff, were asked to complete various questionnaires and had additional HIV testing (blood withdrawals and rapid tests) [23]. Our aim was to understand the experiences of the general community members - without adding burden to existing trial cohort participants [24]. We collected longitudinal qualitative data from a cohort nested in the trial. In this analysis, we report on data collected from households in Arms A and B, where ART was available to all PLHIV [20]. These households offer the opportunity for a contextual understanding of ART management in the context of universal access of ART prior to changes in national guidelines. We use data from households in South Africa that took part in the qualitative cohort to understand people’s experiences of managing income and ART adherence, including their ability to adhere to their treatment plan – i.e., consistent clinic appointment attendance, and taking medications as prescribed (frequency, dosage, and with food/supplementary medication).

### Data collection process

As part of data collection, we used interview guides written in English, but conducted the interviews using local languages comprehendible to participants (Afrikaans, English, or Xhosa). All data collectors were graduate socio-behavioral scientists (including authors AM, LV) who were at least bilingual in the participants’ preferred language and English [24, 25]. A participatory and ethnographic approach was employed to collect data across ‘domains of life’ during multiple interactions with participants to gain a broad understanding of people’s lives and their HIV care and testing experiences [21]. Using a participatory approach, we conducted activities with participants, including drawing kinship maps, mapping household structures, and creating pie charts with yarn showing household income and expenses. The ethnographic approach was carried out through multiple interactions, spending time with, and observing day to day livelihoods of participants [25]. Participants were interviewed at regular intervals (every 2–4 months) and research participants used semi-structured interview guides, structured around central themes. We conducted 3 to 4 group discussions with each of the 89 enrolled households and did at least 1 to 3 one-on-one in-depth interviews among 288 participants (of the overall 370) across the 9 study communities over the period of 18 months (2016–2018) [21]. All interviews were audio-recorded and reflective field notes were taken.

### Sampling procedure

As part of the qualitative cohort in South Africa, we purposefully selected households in the intervention (Arm A and B) communities with members who self-disclosed that they were living with HIV. We purposefully sub-sampled 21 PLHIV from 13 households across the 6 communities. These participants were selected for diversity in terms of age and gender and household composition (multigenerational or extended households, couples, single caregiver households, etc.).

### Data analysis

For our analysis, we used data from the 21 PLHIV in 13 households across the 6 selected communities. All interviews were transcribed verbatim, translated to English, and quality checked. We analysed 25 transcripts using thematic framework [26]. We used a deductive approach, where theme development was informed by a pre-existing framework based on the ART adherence experiences of PLHIV. Author (AM) created the themes and cross-checked with authors LV and PB. We used ATLAS.ti to organise data and to code and identify themes [27].

### Ethics

The study received ethics approval from Stellenbosch University (Ref no. S19/10/227). All participants signed written informed consent in accordance with guidance from the in-country research ethics committee and the consent was continuously confirmed during the implementation of the study. All the names and identifiers of participants were removed, and pseudonyms are used throughout. Prior to discussions, we arranged with participants to find an appropriate and convenient time and confidential space in and around the households to conduct interviews. Although spaces were confined, we often held discussions outside, or at times when other household members were not at home. All data were stored securely. Hard copies of fieldwork notes and consent forms are stored in the locked cabinets at the Desmond Tutu TB Centre. Electronic copies of data and audios are kept on password-protected computers where the access is limited to authorized staff.

## Findings

Ten men and eleven women, with an age range of 18 – 70 years old (median age of 35 years) were included in the analysis. Fifteen participants self-reported that they were ‘adherent to ART’; i.e., regularly attended scheduled appointments with health providers, taking ART as prescribed, but would occasionally miss the dose of ART. Six participants reported that they were not consistently accessing or adhering to ART at the time of the interviews, i.e., that they were often not able to access ART at public health facilities or they were unable to take ART consistently even if they were able to fill their prescriptions. In Table 1 below, we show the demographics of all household members including age, gender, and HIV status of each participant. We also included descriptions of the physical structure of households as an indication of the socio-economic backgrounds of participants.

**Table 1:** Household demographics

Household/ family name (pseudonym)	Name (pseudonym) ^1^	Age	Gender	HIV status	Employment status	Household structure
Norman household	**Betty**	33	Woman	HIV positive	Unemployed	Reconstruction and Development
	**Peter**	30s	Man	HIV positive	Employed	Programme (RDP) house with four rooms
	Poppy Nelson Yolanda **Ludwe**	18 3 13 20s	Man Man Woman Woman	HIV negative Undisclosed Undisclosed Undisclosed	Unemployed Unemployed Unemployed Unemployed	and a bathroom inside and with electricity and water.
Baloyi household	**Nontombi**	43	Woman	HIV negative	Employed	An informal housing unit.
	**Zonke**	28	Woman	HIV positive	Unemployed	(shack) with no amenities.
Gobingca household	**Nolubabalo**	42	Woman	HIV positive	Unemployed	A three room shack dwelling
	**Songezo** Fezile Olothando	17 50s 12	Man Man Woman	HIV negative HIV positive HV negative	Unemployed Employed Unemployed	as tenants at the back of an RDP house owned by another family.
Jacobs household	**Priscilla**	45	Woman	HIV positive	Employed	An old RDP housing unit (two rooms)
	Arlene	25	Woman	Undisclosed	Employed	with an extended informal section
	Yolanda	21	Woman	Undisclosed	Unemployed	built from wood and zinc material.
	Darren	13	Man	HIV negative	Unemployed	
	**Eddie**	33	Man	HIV positive	Employed	
Wislon household	**Joseph**	29	Transgender man	HIV positive	Unemployed	An extended RDP house.
Phaliso household	**Bongiwe**	48	Woman	HIV positive	Unemployed	An RDP house, with electricity,
	Sibusiso	24	Man	HIV negative	Unemployed	water, and bathroom
	Nkosi	20s	Man	Undisclosed	Unemployed	inside. There are two one
	Thuso	26	Man	Undisclosed	Unemployed	room sharks at the back
	**Linda**	19	Woman	HIV positive	Unemployed	rented to tenants.
Mgcina household	Ikho	13	Man	HIV negative	Unemployed	Backyard tenant (shack)
	**Lawrance**	59	Man	HIV positive	Unemployed	at the back
	**Nontobeko**	43	Woman	HIV positive	Unemployed	of an RDP house.
	Lerato	18	Woman	HIV negative	Unemployed	
Dalasile household	Lungani	30s	Man	HIV positive	Employed	An RDP house (two rooms),
	**Khumbuza**	29	Woman	HIV positive	Unemployed	extended with wood and
	**Nomfundiso**	45	Woman	HIV positive	Unemployed	zinc material.
Davidson household	**Dayson**	34	Man	HIV positive	Unemployed	An RDP house (two rooms).
	**Conrad**	70	Man	HIV positive	Unemployed	
Donisi household	**Cathline** **Vuyo** Bongi Kuhle Andisiwe Sihle	51 28 26 19 11 1	Woman Men Men Men Woman Woman	HIV positive Undisclosed Undisclosed Undisclosed Undisclosed Undisclosed	Unemployed Employed Employed Unemployed Unemployed Unemployed	An RDP house (two rooms), extended with wood and zinc building material to be four rooms and a bathroom and running water are accessible outside of the house.
Nofemele household	**Thobela**	39	Man	HIV positive	Employed	An RDP house (two rooms)
	Thabiso Ezile	24 20s	Man Man	HIV negative HIV negative	Unemployed Unemployed	and a one room shack outside.
Mxongo household	**Xolisa** **Nomawethu** Amahle Lihle	52 40s 21 4	Man Woman Woman Man	HIV positive HIV positive HIV negative HIV negative	Employed Employed Unemployed Unemployed	An RDP house (four rooms) with bathroom and water connected outside the house.
Jansen household	**Nwabisa** **Robert**	29 40s	Woman Man	HIV positive Undisclosed	Unemployed Employed	Tenants renting a one room shack in the informal housing unit.

All household members who took part in the discussions are bolded on the table, but only those living with HIV were included in the analysis of how management of income impacts on ART adherence^1^

Eight families lived in basic government-subsidised brick houses – known as RDP (Reconstruction and Development Programme) houses. Three families were tenants renting space in the backyards of RDP houses owned by other individuals. Their backyard informal houses were constructed mostly with zinc and wood. The remaining two families lived in informal housing units, not connected to other formal structures. The average number of occupants on these households was 4, and their relations with one another were complex in that most households were main family houses. People lived with their relatives such as cousins, aunts, nephews, and in-laws. In South Africa, 55% of people live below the poverty line and it is estimated that 14% of residents live in informal settlements which are lower socio-economic status communities [1, 14]. Neighbourhoods like these, including the ones where this study was based, are marked by high levels of unemployment, illiteracy, poor sanitation services, and high levels of crime. Most people in these communities rely on public health services, including for HIV/ART care [1, 28]. We analysed the management of income at the household level while the adherence experiences of PHLIV were analysed at the individual level.

### Household income

We found that living conditions were challenging for families and people were often forced to make difficult decisions to survive. The average income of the 13 households in the study was **±** R3000 ($180) per month. As reported in other resource-constrained settings, participants were able to access resources and incomes from a variety of often unexpected sources [18, 29, 30]. In Table 2, we show that, in addition to more conventional income sources, like wages from casual and part-time work, some families in this study relied on government social grants, renting out unoccupied backyard spaces, and receiving financial assistance from relatives. At times, families would run out of resources before receiving their next income. Many families would opt to borrow money from unregulated lenders, do household chores for neighbours in exchange for cash, or run street vendor businesses to supplement their limited incomes.

**Table 2:** Sources of income

Category	Example
Government social grant	“I don’t have any other reliable sources of income besides the [government social] grant money. I only get disability grant for being sick […] and child support for this three-year-old one [granddaughter]. It’s the only one” (Cathline, 51, Female).
Financial support from relatives	“I don’t even spend money. I am unemployed. Where do I get the money to spend? It’s the people here [neighbours]. Look, you see all the people and everyone here loves me. So here I’m having a lot of food from people around, I don’t worry” (Dayson, 34, Male).
Part-time jobs	“You work and work but sometimes they [employers] say, ‘No don’t come to work. We haven’t got a site [for work] yet’. And [then it] would take the whole week to get paid again while you sit and starve in the township with no pay” (Thobela, 39, Male).
Street vendor businesses	“After I’ve quit that job of looking after an old man, I’ve been selling pork meat ever since. It’s R350 ($20) to stock and the only profit I get is R150 (8$). Then I have to go buy more pork to sell again” (Bongiwe, 48, Female).
Renting out backyard spaces	“They [tenants] pay rent every month end and then they […] go buy their own electricity. Their rent money is separated from electricity because they will say I misuse their money. So I give each of them a slip to go buy electricity themselves and punch it in the box [electricity meter].” (Nomfundiso, 45, Female).
Household chores for neighbours	“If they want their washing [laundry] done they must buy me a cool drink. I won’t go to bed without food whereas there’s something that can feed me […] or just buy electricity. Maybe the others would even give me R50 ($3.5)” (Nolubabalo, 42, Female).
Informal lenders/Loan sharks	“I would borrow money from loan sharks […] say, I’m going to the clinic to fetch my treatment [ARVs]. I don’t want to go to this one [local clinic] here. So, it’s better to borrow [money for transport]” (Nwabisa, 29, Female).

Participants listed routine expenses including food, childcare, preschool, transport, burial policies, electricity, rent, and clothing. With some exceptions, most participants in the study mentioned indirect HIV care-related costs (see Table 3).

**Table 3:** Routine expenses

Category	Example
Basic needs	“First thing I do with money is […] I go buy meat which is high [expensive]. I then take the rest of the remaining money to go buy what I need most which is fish oil, rice and vegetables or something, but the food money is very little […] there are still policies, school stuff” (Betty, 33, Female).
Savings and investments	“We are [part of a] ‘stokvel’ [savings scheme]. We contribute a lot per month and there’s ten of us. I meet the target through business income. You see today is the 3rd [of February] tomorrow I’m contributing, and I already have it” (Khumbula, 29, Female).
Recreational activities	“I would spend money on food but I’m drinking [alcohol] also. You must understand that, and these past few days I’m drinking a lot. I’m stressed. My life alone is a disaster. I have no money and I’m stuck with a shitty job” (Nontombi, 43, Female).
HIV care-related expenses	“With the little money left I would […] every day, I must buy and use pain tablets which are very expensive. I must specifically go and buy at least four to five packets of pain tablets for ARV side effects” (Lawrence, 51, Male).

### Managing decisions on income sharing and expenditure

We found that households had different ways of managing their income. Managing income was determined by decisions people made towards sharing of expenditure, either (a) individually; (b) through ‘selective pooling’ or (c) in an unstructured manner.

For households managing incomes individually, each person in the household would decide on their earned income independently without consulting or sharing their finances with other members of the household. This was common in households without dependents or children. For instance, Dayson (34, male), living with his father who is also living with HIV, noted that:

I support myself, I make sure that I don’t go to bed without food. It’s my fault if I don’t eat. My father goes and borrows money for himself or sometimes would eat at his people [other relatives] where he normally goes (Dayson, 34, Male).

In some households, people decided on income through what we term as ‘selective pooling’. This includes instances where people decided according to their own discretion, to keep a percentage of their earned resources for themselves, while contributing a certain or predetermined amount to the shared expenses of the household. Xolisa (52, male) described how he shared expenses for the household with his wife:

At home what I pay for are clothing accounts and focus on children’s expenses and their school things. Anything related to children depends on me. Groceries, rent, and electricity are handled by my wife (Xolisa, 52, Male).

At times, people would still share their income with others but decisions were not predetermined or discussed before spending. This manner of managing income was unstructured as members were not required and/or expected to contribute but would still cover expenses according to their own discretion. This was often the case when children shared a household with their parents, or when young people shared a household. For example, Khumbuza (29, female), living with her brother and sister explained that:

We don’t decide [on money] but a person buys what someone else couldn’t buy. My brother doesn’t like talking about stuff concerning his money. When he’s about to get paid he just looks what’s there and what’s not there then when he comes back from work he just buys those things (Khumbuza, 29, Female).

These three ways in which income management was decided affected the ability of PLHIV to access and adhere to HIV treatment. The power of deciding on income depended on the relationship people had in the household. Households with ‘pooled’ resources differed from those with individual sources in a sense that they had a strong support system, while those with individual sources were mostly dysfunctional.

### Interaction between managing household income and ART adherence

We found diversity in how household income interacted with the ability of PLHIV to access and adhere to HIV treatment. PLHIV reported that, due to changes in social and economic circumstances often occurring over time, including changes in employment, household composition, and social support structures, they struggled to consistently access and adhere to treatment. We found three ways in which the management of household income interacted with ART access and adherence i.e., (a) the diverting of resources towards substance use activities, (b) the sharing of household resources to support HIV-related care and, (c) prioritising household costs at the expense of ART adherence.

### Diverting income towards substance use activities

At least in 8 of the 13 selected households, members living with HIV reported that, in addition to conventional expenses, they diverted some of their incomes to addictive substance use activities such as buying illicit drugs, which many participants noted would negatively impact their ability to access or adhere to ART. For instance, Dayson (34) was living with his father (71) in an RDP one-roomed house. Both men were living with HIV and depended on the financial support of relatives to ‘get by’. Dayson reported why he could not initiate ART immediately after his HIV diagnosis, despite being eligible for treatment:

When you start using [illicit] drugs then you don’t care about anything. Every [bit of] money you get you spend it on drugs and ignore everything, even your own health, like you ignore taking [ART] medication (Dayson, 34, Male).

In some instances, PLHIV reported that they would spend money on alcohol to ‘numb the pain of knowing about their HIV diagnosis’. Zonke (28, Female), whose main source of income in their household was sex work, was living with her partner. When we asked about HIV treatment, she reported that:

We just buy alcohol and drink. I will drink and get drunk now and tomorrow the stress [of living with HIV] is still there, you see? I’m getting sicker, but I haven’t taken treatment yet (Zonke, 28, Female).

Consuming alcohol would also interrupt the treatment adherence of people who already initiated ART care. Nolubabalo (42, Female) explained to us how her estranged husband, Fezile (living with HIV), would miss prescribed times of taking his ARV doses when he consumed alcohol and would try to ‘catch-up later’.

He would drink alcohol the whole weekend and ignore taking his medication. When he is sober, he would take all the doses he missed over the weekend all at once. But that’s overdose because he’s taking a lot [of pills] in a short time frame (Nolubabalo (42, Female).

In summary, these participants did not explicitly choose to use their resources for substance use activities above spending them on HIV care-related costs. Rather, people would spend their limited income on addictive substances, which negatively affected their ability to initiate or remain on treatment.

### Sharing resources to support HIV care

In some households, various income sources were pooled to support members living with HIV. This included prioritizing food and additional medication that help to ease the side effects of ARVs available at the time. By managing household income in this way, families were able to support treatment adherence. While ART has improved over time and most ARVs currently available are well-tolerated, at the time of data collection some participants noted that they often experienced discomfort or pain, which they attributed to side effects from their ARV medication. For instance, Lawrence and Nontobeko (both living with HIV and ‘getting by’ through the government child support grant they receive for their children) had to put money aside to buy additional pain medicine (over-the-counter). According to them, they needed this medication ‘to ease the ARVs’ side effects’ as experienced by Lawrence:

I get headaches from taking ARVs. Every day I must drink two Grandpas [paracetamol] and pain tablets because without them, I won’t come right [reported hallucinations]. So we take some of the money from the kids’ grant and buy these pills from the chemist (Lawrence, 51, Male).

There were other instances where spending household income on HIV care-related expenses supported the adherence of household members living with HIV. For instance, Nomfundiso who stays with her adult brother and sister explained how her brother, who was the main breadwinner at the time, offered the limited available funds to cover her healthcare costs:

Whenever I ate, I’d just vomit, and I lost energy. My brother was working at the supermarket back then. He insisted on giving me R200 [$13] to go to the [private] doctor. The doctor gave me medication to regain strength and referred me to the [local public] clinic (Nomfundiso, 45, Female).

In this case, the health of the person living with HIV was deemed a priority, and efforts were made to seek care for Nomfundiso, regardless of the financial burden on the household.

In some households, PLHIV were able to generate income while accessing care through the help of relatives. For instance, Bongiwe (48) who worked as a domestic worker would send her daughter, Linda (19) who was also diagnosed with HIV, to fetch her treatment from the clinic while she was at work. Bongiwe explained how that kept her adherent to ART:

Sometimes I send Linda [to the clinic] when I am busy with something thing or when [Linda] is free with no school work to do and even when I just started working she would fetch my medication for me (Bongiwe, 48, Female).

In this case, people were able to balance the act of ‘getting by’ with their health care appointments.

### Competing priorities

Some participants reported that they struggled to balance between attending health services and/or going to work, both of which were deemed to be important for the well-being of PLHIV. For many people who were informally employed or earning minimum wage, there was little flexibility, and taking a leave of absence would compromise their much-needed employment. For instance, Thobela (39), who stayed with his two young-adult brothers and financially supported his extended relatives in the Eastern Cape province, would sometimes forego clinic appointments to go to work as a road construction worker. He explained that:

I was in the Eastern Cape for six months. I don’t remember the year, but I think it was 2014. I took them [ARVs] very badly [infrequently] because when I came back, I quickly got a job. I didn’t have time for the clinic. At some point, I took my clinic card and sent children [to fetch his tablets] but they said [at the clinic] I must go there myself (Thobela, 39, Male).

Thobela described that he wanted to access treatment and he tried to make alternative arrangements to have his medication collected. However, his employment and the responsibility of taking care of the financial well-being of his household took priority while that compromised his clinic attendance and his ability to access ART.

Several PLHIV also reported that they often had additional health-related HIV costs as they had to purchase over-the-counter medication to counter side effects attributed to ART. Costs related to HIV care could, at times, not be met when resources were assigned to other necessities such as food, electricity, and school expenses. For example, Bongiwe (Linda’s mother) explained why Linda could not consistently take ART due to other costs:

We went to fetch Linda’s treatment [from the clinic]. Her medication goes hand-in-hand with *aqueous cream* [body lotion] and tubes for her skin rash. The problem with the clinic is that they run out of stock and they tell us to go buy it at the chemist. How am I supposed to get money to buy that lotion? It’s so expensive. I don’t have money. The money is finished [spent] on food and on her other school stuff (Bongiwe, 48, Female).

Overall, managing income was determined by decisions people made towards sharing of their expenditure. Adherence was complicated by a myriad of factors such as diverting incomes for substance use, employment commitments, and prioritising other basic household expenses over ART related costs.

## Discussion

We reported qualitative data from the HPTN 071 (PopART) trial on the interaction between managing income and ART adherence in the Western Cape, South Africa. We found that PLHIV used their limited resources to buy substances such as drugs and alcohol to numb the emotional challenges of living with HIV. Other household pooled their income to support access and adherence to ART for PLHIV by sharing expenses among members. A third group prioritized other competing expenses in households, such as covering food over ART related costs, which disrupted the ability of PLHIV to access or adhere to ART.

Researchers in low-income settings identified various socio-economic factors, including challenges surrounding household income, as a barrier to ART adherence [9, 21, 22, 30, 31]. Similar to this analysis, studies in South Africa and Uganda found that consistent access to ART was hampered by financial household responsibilities [15, 30, 32]. We found that PLHIV would miss clinic appointments because of work commitments and the need to generate income. However, in some cases, families would facilitate treatment access and adherence for their relatives by resorting to strategies such as collecting their medication while PLHIV were at work.

Qualitative data in low-income settings have shown that when a household is supporting treatment adherence of an individual, there are also additional financial strains experienced by these households [33, 34]. Kibret and colleagues noted that supporting ART adherence includes allocating other household resources to address HIV care-related costs, such as reserving transport fees for PLHIV to attend clinic appointments [30, 34]. Our study found that it was only in few cases where people would reserve resources for HIV care-related costs. For most households, this was not a feasible option as people reported that they had to prioritize basic needs such as food, electricity, and school fees over HIV care-related expenses.

Our study suggests that achieving optimal treatment access and adherence was complicated by multiple factors such as the need to buy and use additional medication to manage taking ART, accessing food before taking medication, and was negatively impacted by the use of illicit drugs and alcohol. However, a systematic review in low and middle-income settings showed that households have the potential to create a health-enabling environment for PLHIV through covering transport fees to the clinic and accompanying relatives when attending HIV care [35, 36]. From our study, a similar approach to support PLHIV would also have been beneficial in that it would encourage PLHIV to attend clinical care.

The strengths of our study include that we drew on longitudinal, in-depth data allowing for thick and nuanced descriptions and a diverse sample of men and women of different ages in a resource-constrained setting. As a limiting factor for this nested-qualitative study, participants could also have viewed the researchers as another type of audience, where telling their stories allowed them to construct themselves and others as particular kinds of moral agents. However, through multiple interactions and observations with participants, researchers were able to learn more about the participants’ lives and gain their trust that made them share their stories in detail. Another limitation include that we only analysed a small sample of the qualitative data and the experiences of PLHIV from the Western Cape province. Therefore, our findings may not be generalizable to other settings, especially if the local social and health system contexts differ substantially.

## Conclusion

Our data shows that PLHIV included in our analysis were able to access and adhere to ART to some extent, although some reported that they could not maintain long-term engagement with health providers and continued adherence of ART. Difficulty in balancing work commitments and clinic appointments, diverting recourses towards substance use, and covering other basic household needs over ART related costs negatively affected the ability of PLHIV to access or adhere to ART. However, households have the potential to improve adherence for PLHIV through prioritising HIV care-related costs. Future HIV/ART programmes could implement treatment delivery or consider ‘after hours’ clinics for patients who are unable to attend care due to work commitments during the day. Overall, mitigating negative impacts of income fluctuation and managing complex trade-offs in households should be included in ART adherence support programmes.

## Data Availability

The data used for the analysis of this project is confidential and can only be made available by the corresponding author and leading investigators of the study upon reasonable request.

## References

[1] Stats SA. Mid-year population estimates. 2021. Available from: www.statssa.gov.za, info@statssa.gov.za.

[2] World Health Organisation. Treat all people living with HIV, offer antiretrovirals as additional prevention choice for people at. ‘substantial’ risk. 2016; Available from: http://www.who.int/mediacentre/news/releases/2015/hiv-treat-all-recommendation/en/.

[3] Meyer-Rath G, Johnson LF, Pillay Y, Blecher M, Brennan AT, Long L (2017). Changing the south african national antiretroviral therapy guidelines: the role of cost modelling. PLoS ONE.

[4] South African National Department of Health. 2019 ART clinical guidelines for the management of HIV in adults, pregnancy, adolescents, children, infants and neonates. 2019.

[5] Sahay S, Reddy KS. Optimizing adherence to antiretroviral therapy. IJMR 2011 D-49. Doi: 10. 4103/097.-5916. 92629. P 22310817; PP. IJMR-134-835. 10.4103/0971-5916.92629 PMC3284093 22310817

[6] Schönnesson LN, Diamond PM, Ross MW, Williams M, Bratt G. Baseline predictors of three types of antiretroviral therapy (ART) adherence: a 2-year follow-up. In: AIDS Care - Psychological and Socio-Medical Aspects of AIDS/HIV. Routledge; 2006. pp. 246–53. 10.1080/09540120500456631 16546786

[7] Simbayi L, Zuma K, Zungu N, Moyo S, Marinda E, Jooste S et al. South African National HIV Prevalence, Incidence, Behaviour and Communication Survey, 2017: Presentation for July 2018 launch. Vol. 2017. 2018. 5–8 p.

[8] Phillips TK, Wilson IB, Brittain K, Zerbe A, Mellins CA, Remien RH (2019). Decreases in self-reported art adherence predict hiv viremia among pregnant and postpartum south african women. J Acquir Immune Defic Syndr.

[9] Azia IN, Mukumbang FC, Van Wyk B (2016). Barriers to adherence to antiretroviral treatment in a regional hospital in Vredenburg, Western Cape, South Africa. South Afr J HIV Med.

[10] Gandhi M, Wang G, King R, Rodrigues WC, Vincent M, Glidden DV (2020). Development and validation of the first point-of-care assay to objectively monitor adherence to hiv treatment and prevention in real-time in routine settings. HHS Public Access AIDS.

[11] Mcinziba A, Wademan D, Viljoen L, Myburgh H, Jennings L, Decloedt E, et al. Perspectives of people living with HIV and health workers about a point-of-care adherence assay: a qualitative study on acceptability. AIDS Care - Psychological and Socio-Medical Aspects of AIDS/HIV; 2023. 10.1080/09540121.2023.2174928 PMC10423296 36781407

[12] Kagee A, Remien RH, Berkman A, Hoffman S, Campos L, Swartz L (2011). Structural barriers to ART adherence in Southern Africa: Challenges and potential ways forward. Glob Public Health.

[13] Steinert JI, Cluver L, Melendez-Torres GJ, Herrero Romero R (2017). Relationships between poverty and AIDS illness in South Africa: an investigation of urban and rural households in KwaZulu-Natal. Glob Public Health.

[14] Nachega J, Uthman O, Mills E, Peltzer K, Amekudzi K, Ouedraogo A. The impact of employment on HIV treatment adherence. Geneva; 2013. Available from: www.ilo.org/publns.

[15] Adeniyi OV, Ajayi AI, Ter Goon D, Owolabi EO, Eboh A, Lambert J (2018). Factors affecting adherence to antiretroviral therapy among pregnant women in the Eastern Cape, South Africa. BMC Infect Dis.

[16] Becker N, Cordeiro LS, Poudel KC, Sibiya TE, Sayer AG, Sibeko LN. Individual, household, and community level barriers to ART adherence among women in rural Eswatini. PLoS ONE. 2020;15(4). 10.1371/journal.pone.0231952 PMC7188206 32343742

[17] Young S, Wheeler AC, McCoy SI, Weiser SD. a review of the role of food insecurity in adherence to care and treatment among adult and pediatric populations living with HIV and AIDS. AIDS and Behavior. 2014;18(0 5):505–15. Available from: https://pubmed.ncbi.nlm.nih.gov/23842717/. 10.1007/s10461-013-0547-4 PMC3888651 23842717

[18] Campbell L, Masquillier C, Thunnissen E, Ariyo E, Tabana H, Sematlane N et al. Social and structural determinants of household support for ART adherence in low- and middle-income countries: a systematic review. Int J Environ Res Public Health. 2020;17(11). 10.3390/ijerph17113808 PMC7312869 32471153

[19] Mudzengi D, Sweeney S, Hippner P, Kufa T, Fielding K, Grant AD (2017). The patient costs of care for those with TB and HIV: a cross-sectional study from South Africa. Health Policy Plann.

[20] Hayes R, Ayles H, Beyers N, Sabapathy K, Floyd S, Shanaube K et al. HPTN 071 (PopART): Rationale and design of a cluster-randomised trial of the population impact of an HIV combination prevention intervention including universal testing and treatment-a study protocol for a cluster randomised trial. 2014; Available from: http://www.trialsjournal.com/content/15/1/57. 10.1186/1745-6215-15-57 PMC3929317 24524229

[21] Seeley J, Bond V, Yang B, Floyd S, MacLeod D, Viljoen L et al. Understanding the time needed to link to care and start ART in seven HPTN 071 (PopART) study communities in Zambia and South Africa. AIDS and Behavior. 2019;23(4):929–46. Available from: 10.1007/s10461-018-2335-7. 10.1007/s10461-018-2335-7 PMC6458981 30415432

[22] Hoddinott G, Myburgh H, de Villiers L, Ndubani R, Mantantana J, Thomas A (2018). Households, fluidity, and HIV service delivery in Zambia and South Africa - an exploratory analysis of longitudinal qualitative data from the HPTN 071 (PopART) trial. J Int AIDS Soc.

[23] Hayes RJ, Donnell D, Floyd S, Mandla N, Bwalya J, Sabapathy K (2019). Effect of universal testing and treatment on hiv incidence — HPTN 071 (PopART). N Engl J Med.

[24] Viljoen L, Ndubani R, Bond V, Seeley J, Reynolds L, Hoddinott G. Community narratives about women and HIV risk in 21 high-burden communities in Zambia and South Africa. International Journal of Women’s Health. 2017;9:861–70. Available from: https://pubmed.ncbi.nlm.nih.gov/29238230/. 10.2147/IJWH.S143397 PMC5716400 29238230

[25] de Villiers L, Thomas A, Jivan D, Hoddinott G, Hargreaves JR, Bond V (2020). Stigma and HIV service access among transfeminine and gender diverse women in South Africa – a narrative analysis of longitudinal qualitative data from the HPTN 071 (PopART) trial. BMC Public Health.

[26] Attride-Stirling J (2001). Thematic networks: an analytic tool for qualitative research. Qualitative Res.

[27] Friesie S. Qualitative Data Analysis with ATLAS.ti. 2019.

[28] Centre for Social Development in Africa (CSDA). Poverty, inequality and social exclusion in South Africa. Natl Dev Agency. 2019;19–64.

[29] Masquillier C, Wouters E, Mortelmans D, le Roux Booysen F (2015). The impact of community support initiatives on the stigma experienced by people living with HIV/AIDS in South Africa. AIDS Behav.

[30] Kibret S (2018). The effect of HIV/AIDS on household’s healthcare expenditure and income in Addis Ababa: a propensity score matching approach. HIV and AIDS Review.

[31] Merten S, Kenter E, McKenzie O, Musheke M, Ntalasha H, Martin-Hilber A (2010). Patient-reported barriers and drivers of adherence to antiretrovirals in sub-saharan Africa: a meta-ethnography. Trop Med Int Health.

[32] Nanfuka EK, Kyaddondo D, Ssali SN, Asingwire N. Social capital and resilience among people living on antiretroviral therapy in resource-poor Uganda. PLoS ONE. 2018;13(6). 10.1371/journal.pone.0197979 PMC5995438 29889849

[33] Bergmann JN, Wanyenze RK, Stockman JK. The cost of accessing infant HIV medications and health services in Uganda. AIDS Care - Psychological and Socio-Medical Aspects of AIDS/HIV. 2017;29(11):1426–32. Available from: 10.1080/09540121.2017.1330531. 10.1080/09540121.2017.1330531 28521509

[34] Negin J, Randell M, Raban MZ, Nyirenda M, Kalula S, Madurai L (2017). Health expenditure and catastrophic spending among older adults living with HIV. Glob Public Health.

[35] Campbell L, Masquillier C, Thunnissen E, Ariyo E, Tabana H, Sematlane N et al. Social and structural determinants of household support for ART adherence in low- and middle-income countries: a systematic review. Int J Environ Res Public Health. 2020;17(11). 10.3390/ijerph17113808 PMC7312869 32471153

[36] Masquillier C, Wouters E, Mortelmans D, Van Wyk B, Hausler H, Van Damme W (2016). HIV/AIDS competent households: Interaction between a health-enabling environment and community-based treatment adherence support for people living with HIV/AIDS in South Africa. PLoS ONE.

